# Gleason 6 prostate cancer: That which cannot be named

**DOI:** 10.3389/fonc.2022.1073580

**Published:** 2022-12-05

**Authors:** Harry B. Burke

**Affiliations:** Department of Medicine, Uniformed Services University of the Health Sciences, Bethesda, MD, United States

**Keywords:** prostate cancer, low-grade cancer, cancer diagnosis, lethality, diagnosis, Gleason score, Gleason grade

There is an ongoing debate regarding whether low-grade cancers, such as Gleason score 6 prostate cancer, should be called cancer. For example, Laura Esserman and colleagues (1), suggest that it should not be labeled cancer. They believe that “minimal risk lesions should not be called cancer.” (p. 1689) “For prostate cancer, low-volume lesions with low Gleason scores have a low risk of causing death within an intermediate period. By doing so [not calling the tumors cancer] and reliably categorizing these lesions with low risk of morbidity or mortality, the burden of therapy can be eliminated in many cases.” They proposed calling low risk prostate cancer “indolent lesions of epithelial origin (IDLE).” (p. 1689)

Carter et al. (2), agreed that there are problems with calling the Gleason score 6 prostate cancer. They stated that, “fear of dying as a result of cancer surely plays a role in decisions to proceed with treatment among men with Gleason 6 tumors.” (2) (p. 4294) Furthermore, they suggested that fear drives overtreatment; that men who would not die of prostate cancer are deciding to be treated. “The extent of overtreatment is caused by fear of death resulting from the cancer.” (2) (p. 4294) They suggested that the problem is the name “Gleason score.” They proposed retaining the name cancer but changing the nomenclature from Gleason scores to five Prognostic Grade Groups (which are based on the Gleason system), with the Gleason 6 now called Grade Group 1 (GG1) prostate cancer. “It is hoped that this [removing the Gleason 6 label] will alleviate some of the fear associated with a diagnosis of Gleason score 6 ‘cancer’ and give patients a more realistic perspective regarding their prognosis.” (2) (p. 4296) In essence, they are saying that patients are afraid of the Gleason score 6 because they believe that cancer means death (6 is more than half way to 10), that this belief is making patients do things they should not do, and that if the label Gleason score is gone, then patients will feel better about their prostate cancer. Unfortunately, renaming the histology does not solve the problem because, adjacent to the histology, are the words prostate cancer.

Ahmed et al. (3) agreed with Esserman et al.’s assertion that we should reclassify Gleason 6 prostate cancer as noncancer. “We believe that small low-grade Gleason pattern lesions, which are current designates as prostate cancer, could be regarded as non-malignant.” (3) (p. e509) This is justified by the idea that not all prostate cancers are destined to progress. “If we could accurately identify men with Gleason pattern 3 lesions in isolation, these men would be very likely to be at much lower (possibly negligible) risk of death from prostate cancer than men previously attributed a Gleason pattern 3 diagnosis of cancer. If this situation came to pass, we might be in a position to reclassify exclusive Gleason pattern 3 lesions to a term that substitutes the word cancer for something else, such as IDLE (3). Such a term would seem to be appropriate; if low-volume, low grade lesions were reclassified as non-cancer or IDLE lesions and this change met with widespread professional acceptance, the immediate implications for clinical practice would be profound.” (3) (p. e515)

Recently, Eggener et al. (4) argued for reclassifying Gleason score 6 tumors as noncancer. They asserted that men with a severity of illness Gleason score 6 on biopsy have a cancer-related death rate that approaches 0% even in the absence of treatment. They went on to say that, because these men will not die of prostate cancer, they do not have prostate cancer. Since they do not have prostate cancer, their diagnosis should be changed to one that does not contain the word “cancer.”

In their rebuttal, Epstein and Kibel (5) ask a key question; if the low-grade cancer is not treated, would it extend out of the prostate resulting in a decreased cure rate? They point out that more than 50% of the men with low risk prostate cancer and who chose active surveillance, receive treatment. They conclude that the argument that most men with a Gleason score 6 who are not treated will not die of their disease is not valid because most men are treated. In support of this argument, a recent population-based study from Sweden (6) modeled the 30-year outcomes of men with prostate cancer diagnosed from between 1992 and 2014 who were managed by active surveillance. It found that, in men with a Gleason score 6 who were diagnosed before age 70, 59.8% received either radical prostatectomy or radiation therapy. In men with a Gleason score 6 score who were diagnosed at or after age 70, 28.5% received radical prostatectomy or radiation therapy. Furthermore, the rate of prostate cancer death of the men with a Gleason score 6 who were diagnosed at age 55 years, and who died of prostate cancer before age 85, was 1 in 11 for very low-risk men and 1 in 8 for low-risk men. Thus, it is unlikely that if none of the men with a Gleason score 6 received any treatment there would be an almost 0% rate of death from prostate cancer.

It should be noted that Esserman (1), Ahmed (3), and Eggener (4) present the Gleason score as if it is the only important severity of illness prognostic factor, when, in fact, other factors must be considered when predicting at diagnosis what will happen to the patient. These factors include age, clinical stage, PSA, number of cores and number positive, cancer length, and prostate volume (6). Furthermore, Epstein and Kibel (5) point out that, in the future, new molecular prognostic factors will be discovered and integrated into an assessment of a patient’s severity of illness at diagnosis.

It is clear that a patient with a Gleason score 6 has prostate cancer. “Morphologically and genetically, Gleason score 6 is cancer with the ability to invade tissues.” (2) (p, 4294) Furthermore, “GG1 is cancer that often looks like higher-grade prostate cancer microscopically and is invasive with a lack of basal cells, infiltration into the prostatic stroma, and frequent perineural invasion.” (5) (p. 3107) The Gleason scoring system was designed to be, and is, prognostic. Gleason and Mellinger (7), in their 1974 paper “Prediction of prognosis for prostatic adenocarcinoma by combined histological grading and clinical staging,” stated that, “Histologic grading of prostatic adenocarcinoma contributes mortality rate prediction information in addition to that provided by clinical staging of the tumor.” (p. s138)

Fundamentally, the argument by Esserman (1), Ahmed (3), and Eggener (4) against a Gleason score 6 tumor being named cancer depends on the idea that the diagnosis of cancer should be determined by a biopsy-based prediction of the tumor’s lethality. The rationale for saying that a Gleason score 6 is not cancer is that, because it will not be lethal, it is not cancer. In other words, the diagnosis of cancer rests on a prediction of the patient’s ultimate outcome.

Clearly, those who promote the idea that a Gleason score 6 tumor is not prostate cancer have confounded diagnosis with prognosis. One cannot use prognostic factor information to deny the existence of a diagnosis.

Rejecting that a cancer is a cancer has at least three important consequences. First, using a Gleason score to diagnose prostate cancer would have profound consequences for medicine because it would be using a severity of illness prognostic factor as if it was a diagnostic factor (8). This would call into question the very nature of diagnosis.

Second, hiding from patients with a Gleason score 6 prostate cancer the fact that they have prostate cancer would have important ramifications for patients. A diagnosis establishes a patient’s right to medical treatment (9). Removing the word cancer may eliminate the patient’s right to cancer treatment because there is no longer a medical indication for treatment. Thus, under the view that these patents do not have cancer, if patients wanted a cancer treatment, they would not be allowed to have it. Furthermore, “…undertreatment of prostate cancer and a missed opportunity for cure in those who would benefit is a real risk of relabeling a cancer as noncancer.” (2) (p. 4294) Finally, Epstein and Kibel (5) address patient fear and anxiety. They state that men have become comfortable with active surveillance and that, in conjunction with their five Grade Group nomenclature (which is based on the Gleason system), low risk disease has lost its ability in instill fear in patients.

Third, taking cancer decision making away from patients would have important social implications. It would represent a return to paternalism. It would legitimize the idea that patients do not have the right to make decisions regarding their disease. It would violate the ethical principle of patient autonomy which states that a patient has the right to self-governance and self-determination. As Childress pointed out, “…the patient’s own health and value systems are at stake, he or she should have ‘moral authority’ superior to the physician’s” (9) (p. 23). Thus, if we were to subvert the diagnosis of cancer, we would deny patients’ right to autonomy and we would reestablish the paternalistic control of patients.

In conclusion, it is incorrect to assert that a man with biopsy-diagnosed prostate cancer and a Gleason score 6 pattern does not have prostate cancer. The correct strategy is to acknowledge that the patient has prostate cancer, to combine the Gleason score 6 with other severity of illness at diagnosis prognostic factors, to incorporate that knowledge into shared decision making, and to continue to work to discover molecular biomarkers (10) that can accurately distinguish between non-lethal and lethal prostate cancer.

## Author contributions

The author confirms being the sole contributor of this work and has approved it for publication.

## Funding

DHA-USU Patient Safety Academic Collaborative.

## Disclaimer

The views expressed in this article do not necessarily represent the views of the U.S. Government, the Department of Defense, or the Uniformed Services University of the Health Sciences.

## Conflict of interest

The author declares that the research was conducted in the absence of any commercial or financial relationships that could be construed as a potential conflict of interest.

## Publisher’s note

All claims expressed in this article are solely those of the authors and do not necessarily represent those of their affiliated organizations, or those of the publisher, the editors and the reviewers. Any product that may be evaluated in this article, or claim that may be made by its manufacturer, is not guaranteed or endorsed by the publisher.
